# Ecological health evaluation of rivers based on phytoplankton biological integrity index and water quality index on the impact of anthropogenic pollution: A case of Ashi River Basin

**DOI:** 10.3389/fmicb.2022.942205

**Published:** 2022-08-26

**Authors:** Zhenxiang Li, Chao Ma, Yinan Sun, Xinxin Lu, Yawen Fan

**Affiliations:** ^1^College of Life Science and Technology, Harbin Normal University, Harbin, China; ^2^Key Laboratory of Biodiversity of Aquatic Organisms, Harbin Normal University, Harbin, China

**Keywords:** phytoplankton biological integrity index, water quality index, Ashi River Basin, anthropogenic activity, water pollution

## Abstract

Based on the phytoplankton community matrices in the Ashi River Basin (ASRB), Harbin city, we developed an evaluation method using the phytoplankton index of biotic integrity (P-IBI) to evaluate ecological health while investigating the response of P-IBI to anthropogenic activities. We compared the effectiveness of P-IBI with that of the water quality index (WQI) in assessing ecological health. Between April and October 2019, phytoplankton and water samples were collected at 17 sampling sites in the ASRB on a seasonal basis. Our results showed that seven phyla were identified, comprising 137 phytoplankton species. From a pool of 35 candidate indices, five critical ecological indices (Shannon–Wiener index, total biomass, percentage of motile diatoms, percentage of stipitate diatom, and diatom quotient) were selected to evaluate the biological integrity of phytoplankton in the ASRB. The ecological status of the ASRB as measured by the P-IBI and WQI exhibited a similar spatial pattern. It showed a spatial decline in ecological status in accordance with the flow of the river. These results highlighted that P-IBI was a reliable tool to indicate the interaction between habitat conditions and environmental factors in the ASRB. Our findings contribute to the ecological monitoring and protection of rivers impacted by anthropogenic pollution.

## Introduction

In recent years, the water pollution caused by anthropogenic sewage discharges, agricultural runoff, and industrial wastewater discharges has adversely impacted the health of aquatic ecosystems in numerous rivers and lakes (Griffiths et al., 2018; Xu et al., 2018). The current methods for assessing the health of the aquatic ecosystem are mainly based on water quality indicators and aquatic organisms. These include the water quality index (WQI; De La Mora-Orozco et al., 2017), biological integrity index (IBI; Wu et al., 2019), and species diversity index (SDI; Meng et al., 2020). A healthy aquatic ecosystem must have high-quality biological integrity before it can be sustainably used and developed (Scanlon et al., 2007).

Biological integrity refers to the ability of a biotic community to maintain structural equilibrium and adapt to environmental changes (Souza and Vianna, 2020). Karr (1981) assessed the ecological conditions of aquatic groups based on 12 attributes linked to the species composition and ecological structure of fish communities. This was the initial approach for assessing biological integrity, and it has been expanded and modified on an ongoing basis (Cui et al., 2018). Assessing biological integrity is a crucial method for assessing the status of ecosystems, and biological integrity indices (IBIs) play a key role in global water resource management (Zhu et al., 2019). Current research on IBIs mainly focuses on fish (Cooper et al., 2018), macroinvertebrates (Wahl et al., 2019), plankton (Zhang et al., 2019), and bacteria (Li et al., 2017, 2018). These aquatic biological community structures are important markers of water pollution and eutrophication; therefore, they are often used to evaluate the damage to and health of aquatic ecosystems (Pereira et al., 2018). Phytoplankton rapidly respond to environmental changes, and their community structure accurately reflects the short-term effects of anthropogenic and natural disturbances on aquatic ecosystems (Busseni et al., 2019). The composition, abundance, biomass, and community stability of phytoplankton are widely used as important indicators of environmental change (Marvaetal et al., 2014). The discharge of pollutants decreases phytoplankton diversity and community structure stability in rivers. In addition, it substantially impacts the biological integrity of phytoplankton and the ecological service functions of river ecosystems (Inyang and Wang, 2020; Hu et al., 2022). Phytoplankton, functioning as producers in the biological chain, are the most sensitive to changes in the river environment; therefore, they are widely used to evaluate changes in aquatic ecology (Amorim and Moura, 2021). The phytoplankton biological integrity index (P-IBI) has received less attention than other IBIs based on fish and macroinvertebrates. Although it has been used to assess river ecosystems in recent years, few studies have focused on its application to assess the impact of anthropogenic activities on river ecosystems (Lin et al., 2021; Wan et al., 2021). Therefore, it is necessary to conduct further research on the potential of phytoplankton to assess the impact of anthropogenic activities on the biological integrity of river ecosystems.

In recent years, rivers in Heilongjiang Province have been polluted to varying degrees owing to the acceleration of industrialization and urbanization, with the Songhua River Basin being heavily polluted. The Ashi River is the main tributary to the southern bank of the Songhua River. The reserve water supply for Harbin is sourced from the Xiquanyan Reservoir upstream of the Ashi River. In recent years, water pollution in the basin has become an increasingly serious concern in light of the steady expansion of the regional economy and grain production. During the wet season, a large quantity of pesticides and fertilizer residues enters the Ashi river combined with surface runoff from the extensive farmland in the middle reaches of the Ashi River Basin (ASRB) (Chen et al., 2022). The region downstream of the ASRB is densely populated, with land use dominated by cities and towns that are severely impacted by anthropogenic activities. There are several small and medium-sized enterprises in the area, and industrial effluent discharge is typically the primary source of pollution (Zhao et al., 2020b). The water quality of the Songhua River, the critical control river of the Harbin region, is gravely threatened by the deterioration of the water quality of the Ashi River, which has destroyed the ecological balance.

In this study, we established a P-IBI method to assess the health status of the ASRB aquatic ecosystem. The main purposes of this study were as follows: (1) to establish the P-IBI of the ASRB under the influence of anthropogenic activities and (2) to effectively explain the water quality and temporal and spatial distribution patterns of the ASRB. In addition, to evaluate the performance of P-IBI, we compared P-IBI evaluation results with those of a water quality index (WQI). We hypothesize that the P-IBI assessment standard can effectively represent the water quality status of the ASRB and is consistent with the performance of the WQI assessment standard. Therefore, we also aim (3) to reveal the relationship between P-IBI and environmental factors. Our research is beneficial to local water resource management, including the formulation of associated control policies, and it makes specific contributions to the development of P-IBIs.

## Materials and methods

### Study sites

**FIGURE 1:**
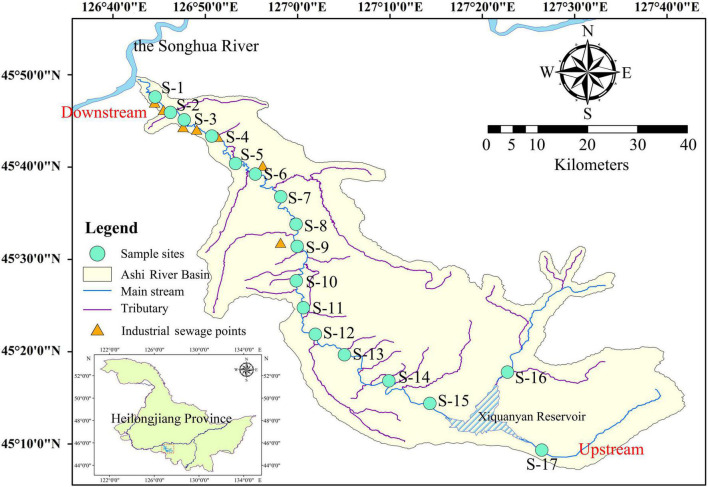
Map of the Ashi River Basin (ASRB) illustrating the sampling sites for this study.

The Ashi River is a primary tributary to the Songhua River on its right bank. It is situated only 80 km from Harbin city. The study area was located between 126°43′ and 127°36′ E longitude and 45°08′ and 45°50′ N latitude (a total distance of 213 km) (Figure 1 and Supplementary Table 1). The Xiquanyan Reservoir is a connected reservoir upstream of the main ASRB. The climate is characterized by a temperate continental monsoon, with subfreezing temperatures from November to April. The annual precipitation in the basin is 580–600 mm, and the annual average temperature is 3.6°C. In this study, 17 sampling sites were selected along the main channel of the river. Samples were collected every 3 months from April to October 2019 to represent the spring, summer, and autumn seasons. We were unable to collect all samples at certain sites owing to heavy precipitation in the spring and summer.

### Water sampling and processing

At each sampling site, surface water samples (0–0.5 m) were collected and subjected to phytoplankton and physicochemical analyses in triplicate. Phytoplankton samples were collected in glass bottles (1 L), immediately fixed with 3% acid-Lugol’s solution, and stored in a dark room at 20°C until further processing. Water temperature (WT), dissolved oxygen (DO), conductivity (Cond.), pH, and turbidity (Tur.) were recorded using a multi-parameter water quality analyser (YSI ProPlus, YSI, United States). Total phosphorus (TP, GB/T11893-89), total nitrogen (TN, HJ 636-2012), chemical oxygen demand (COD_*Mn*_, GB/T11892-89), and biochemical oxygen demand (BOD_5_, HJ 50-2009) were the environmental factors analyzed in the laboratory.

### Plankton identification

Phytoplankton were examined at ×400 magnification (× 10 eyepiece and × 40 objective) using an Olympus microscope (Optec B302, Chongqing, China) with a 0.1 mL plankton counting chamber (Yuan et al., 2018). Most phytoplankton samples were identified at the species level, and their abundance was expressed as ind. L^–1^. The publication “Freshwater algae in China: systems, classification and ecology” (Hu and Wei, 2006) was used to identify phytoplankton.

### Establishing the phytoplankton index of biotic integrity assessment system

In accordance with previous studies (Lacouture et al., 2006), the steps to establish an IBI system are as follows:

#### Determination of reference and impaired points

In this study, the WQI was used in conjunction with the results of an *in situ* investigation, and sampling sites were divided into two groups, namely, reference and impaired points. The points were evaluated using the WQI method (Pesce and Wunderlin, 2000; Kannel et al., 2007; Koçer and Sevgili, 2014; Sun et al., 2016). The equation used for calculating WQI was as follows:


(1) $$WQI=\frac{\sum_{i=1}^{n}C_{i}P_{i}}{\sum_{i=1}^{n}P_{i}}$$

where *n* is the total number of parameters included in the study, *C*_*i*_ is the normalized value of parameter *i*, and *P*_*i*_ is the weight of parameter *i*. The minimum value of *P*_*i*_ was 1, and the maximum weight assigned to parameters that affect water quality was 4; these values have been verified in previous publications (Supplementary Table 2; Pesce and Wunderlin, 2000; Kannel et al., 2007; Koçer and Sevgili, 2014; Sun et al., 2016). The WQI ranged from 0 to 100, with high values indicating good water quality. Based on the WQI scores, water quality was categorized into five grades as follows: excellent (91–100), good (71–90), moderate (51–70), low (26–50), and bad (0–25) (Wu et al., 2019). The seasonal WQI values of each sampling site were averaged to determine the final WQI value.

Since there are numerous villages and agricultural land along the ASRB and many factories in its middle and lower reaches, it is difficult to obtain a clean water reference point for the development of a P-IBI unaffected by anthropogenic activities (Stoddard et al., 2006). Such a point should adhere to four conditions: (1) a good or excellent annual average WQI index evaluation grade; (2) high vegetation coverage, no overdevelopment of the shoreline, no sand fields, no wharf, and no random reclamation on the bank slopes; (3) no garbage stacking on the bank slope, no garbage floating on the water surface, no peculiar odor, and a relatively clear water body; and (4) a good phytoplankton diversity and phytoplankton abundance at less than 2 × 10^6^ ind./L (Li et al., 2014, 2020).

#### Biological indicators screening

The biological indicators for establishing IBI need to be followed by the distribution range, discriminant ability, and correlation tests, and the remaining indicators were used to construct the IBI assessment system (Lacouture et al., 2006). To perform the distribution range test, it was necessary to calculate the values of candidate biological indicators based on the data at the reference and impaired points. Then, the responses of the candidate biological indicators to anthropogenic disturbances were analyzed to identify those that increased or decreased unidirectionally. The discrimination ability test initially screened strong ability indicators by evaluating the extent of boxplot overlap between the reference and impaired points. In cases where the correlation coefficients between a pair of indicators in Spearman’s correlation analysis show |r| > 0.75, one of the indicators should be omitted to avoid redundancy (Maxted et al., 2006).

#### Index of biotic integrity calculation and assessment grading standards

The ratio method was applied to unify the indicator dimensions (Zhang et al., 2019). The scores for the ratio method were calculated in different ways. For indicators where the values decreased as anthropogenic intervention increased, we used:


(2) $$S=\left(P-P_{min}\right)/\left(P_{95\%}-P_{min}\right)$$

where P refers to the indicator value and *P*_95%_ is the 95% percentile of this indicator for all points. *P*_*min*_ is the minimum value of a specified indicator. For indicators where the values increased as anthropogenic intervention increased, we used:


(3) $$S=\left(P_{max}-P\right)/\left(P_{max}-P_{5\%}\right)$$

where *P*_*max*_ was the maximum value of a specified indicator and *P*_5%_ was the 5% quantile for all points. The P-IBI of each sampling site equaled the average of S for all the selected indicators at that point, which was given as follows:


(4) $$P-IBI=\frac{1}{t}\sum_{j=1}^{n}S_{j}$$

where *S*_*j*_ was the *S* value of the *j*_*th*_ point and *t* was the number of samples collected at each site. Finally, the 95% quantiles of P-IBI for all sampling sites were used as the lower limit for delineating the excellent level. If the P-IBI of the sampling sites was greater than this value, the sampling point was healthier and less impacted by anthropogenic disturbances. The IBI range that was less than the lower limit for delineating the ‘excellent’ grade was divided into four categories: “good,” “fair,” “poor,” and “extremely poor.”

### Statistical analyses and tools

Microsoft Excel 2016 was used for data processing, partial biological index calculations, and box plots. Shannon–Wiener, Margalef, and Pielou index calculations were performed using R 3.6.3, and inverse distance weighting interpolation within the study area was performed using ArcGIS 10.2. An independent-samples *t*-test was conducted to evaluate differences in environmental data and was performed using SPSS 20.0 for Windows (SPSS Inc., Chicago, IL, United States).

Prior to performing a multiple regression analysis, the collinearity among the independent variables should be diagnosed according to the eigenvalues, condition index (CI), variance proportion, tolerance, and variance inflation factor (VIF) (Liu et al., 2010). P-IBI was used as the dependent variable in an MLR (with forwarding selection) where partial regression coefficients for the independent variables were selected using a *t*-test at a significance level of 0.05 using SPSS 20.0 software (SPSS Inc., Chicago, IL, United States).

## Results

### Species and abundance of phytoplankton

**FIGURE 2:**
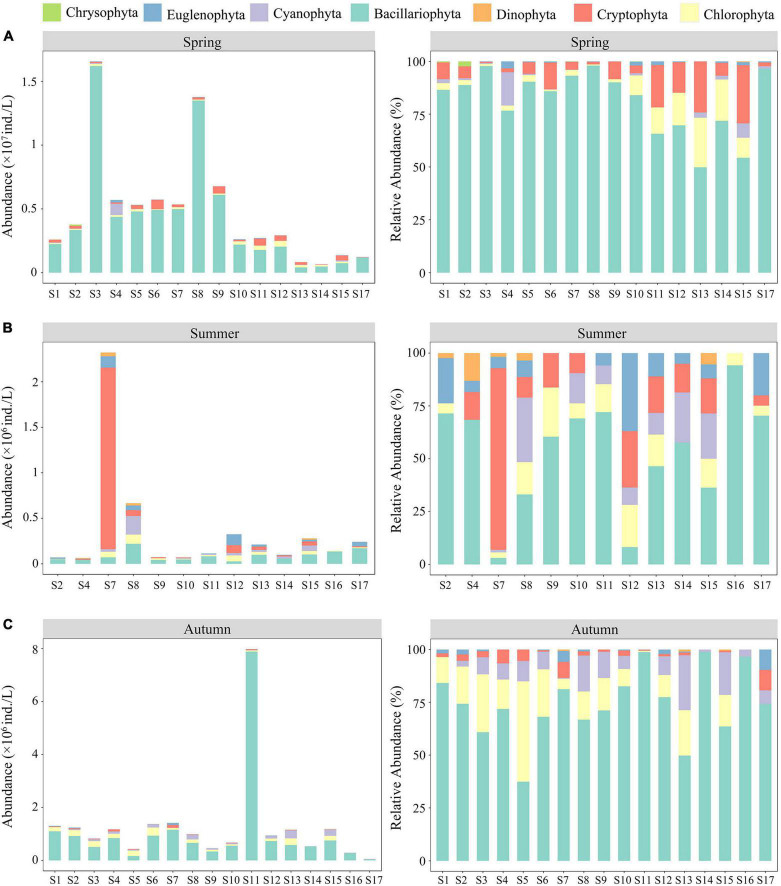
Abundance and relative abundance of phytoplankton at various sampling sites: **(A)** spring; **(B)** summer; and **(C)** autumn.

In this study, 137 species of phytoplankton belonging to 7 phyla, 10 classes, 15 orders, 26 families, and 62 genera were identified throughout the spring, summer, and autumn of 2019. Phytoplankton species were mainly composed of Bacillariophyta (65 species, 47.45%) and Chlorophyta (40 species, 29.20%), followed by Cyanobacteria (14 species, 10.22%) and Euglenophyta (11 species, 8.03%), whereas the proportions of Cryptophyta, Dinophyta, and Chrysophyta were lower than those of the aforementioned phyla. Overall, the number of phytoplankton species in autumn (97) was much higher than that observed in spring (74) and summer (76). The abundance and relative abundance of phytoplankton at various sampling sites during the three seasons are shown in Figure 2. The temporal and spatial distributions of phytoplankton abundance in the ASRB exhibited distinct differences. In terms of time, the abundance of phytoplankton in spring (4.48 × 10^6^ ind./L) was much greater than that in summer (3.60 × 10^5^ ind./L) and autumn (1.29 × 10^6^ ind./L), with the abundance of phytoplankton declining from spring to summer and increasing from summer to autumn. Except for S7, S8, and S12 in the summer, diatoms were the predominant organisms at each sampling site during the study period. In a spatial context, upstream phytoplankton abundance was determined to be the lowest, whereas downstream phytoplankton abundance was determined to be the highest. Simultaneously, during summer and autumn, the dominant position of diatoms from upstream to downstream gradually declined.

### Environmental factors of Ashi River Basin

Except for TN, other environmental factors exhibited statistically significant seasonal differences (*P* < 0.05) (Table 1). The WT ranged from 3.7 to 26.0°C, reaching its highest value in summer. The pH values of the water ranged from 7.16 to 9.14, making it alkaline. Conductivity values ranged from 78.3 μS/cm (S15 in autumn) to 649 μS/cm (S2 in spring). TN values were similar in spring and summer, and the highest TN concentrations were recorded at S2 (2.33 mg/L). The highest TP concentrations were recorded in summer, ranging from 0.01 to 1.08 mg/L. COD_*Mn*_ concentrations ranged from 3.88 to 12.86 mg/L, with the highest value recorded at S2 in summer, and the lowest value recorded at S17 in spring. DO values ranged between 1.40 and 16.44 mg/L. The highest BOD_5_ value was recorded at S4 (10.10 mg/L) in spring, and the lowest values were recorded at S10 and S11 (0.30 mg/L) in spring. The Tur. ranged from 7.5 NTU (S14 in autumn) to 155.5 NTU (S9 in summer).

**TABLE 1:** Maximum, minimum, and mean values of envorinment factors in the ASRB.

		Season			T-text	
		Spring	Summer	Autumn	Spring-Summer	Summer-Autumn
WT	Max	17.9	26	13.6	*P* < 0.05	*P* < 0.05
	Min	10	18.7	3.7		
	Mean	15.56	22.72	9.34		
pH	Max	9.14	7.08	8.15	*P* < 0.05	*P* < 0.05
	Min	7.16	6.66	7.31		
	Mean	8.4	6.91	7.81		
Cond.	Max	694	136	338.7	*P* < 0.05	*P* < 0.05
	Min	121.6	78.3	96.6		
	Mean	366.49	107.61	209.22		
TN	Max	2.33	0.9	0.83	*P* > 0.05	*P* < 0.05
	Min	0.12	0.14	0.41		
	Mean	0.95	0.78	0.59		
TP	Max	0.76	1.08	0.56	*P* < 0.05	*P* < 0.05
	Min	0.01	0.06	0.01		
	Mean	0.21	0.44	0.14		
COD_*Mn*_	Max	10.9	12.86	12.84	*P* < 0.05	*P* < 0.05
	Min	3.88	5.65	4.75		
	Mean	6.48	10.7	8.39		
DO	Max	15.1	10.1	16.44	*P* < 0.05	*P* < 0.05
	Min	12.3	1.4	9.6		
	Mean	13.36	8.05	12.81		
BOD_5_	Max	10.1	9.8	1.9	*P* < 0.05	*P* < 0.05
	Min	6.1	0.9	0.3		
	Mean	7.59	3.3	0.98		
Tur.	Max	89.2	155.5	31.3	*P* < 0.05	*P* < 0.05
	Min	16.8	18.8	7.5		
	Mean	48.35	81.25	14.72		

### Water quality assessments based on water quality index

**FIGURE 3:**
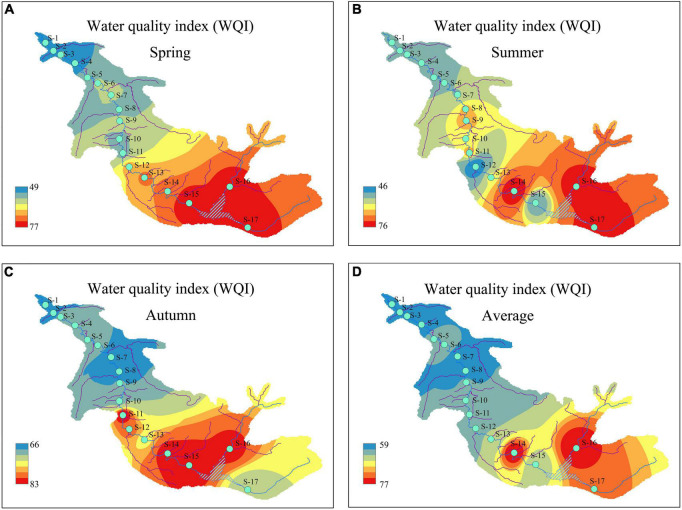
Temporal and spatial distributions of water quality index (WQI) during the study period: **(A)** spring; **(B)** summer; **(C)** autumn; and **(D)** average.

During the study period, the WQI had distinct patterns of temporal and spatial changes (Figure 3). From a seasonal perspective, the WQI was highest in autumn (77), followed by spring (64) and summer (61). Compared with spring and summer, the WQI of autumn showed a significant change (*P* < 0.01), indicating that the water quality in autumn (good) was significantly better than that in spring and summer (moderate). From a spatial perspective, the WQI of the sampling sites surrounding the reservoir upstream of the river had the highest value during each season. Simultaneously, the changes in the WQI manifested as a gradual upstream-to-downstream decline in the river. Similar WQI spatial patterns were observed among seasons, indicating that the water quality of the ASRB was best upstream and gradually deteriorated downstream. According to the WQI classification standard, 12 sampling sites (S1–S12) in the ASRB were classified as “moderate,” and 5 sampling sites (S13–S17) were classified as “good.”

### Phytoplankton index of biotic integrity calculations and health assessment of river ecosystems

The primary sources of water pollution in the ASRB were industrial effluent and agricultural runoff, and single-factor evaluation methods could not accurately determine the relative severity of water pollution. We used the WQI to determine the water quality at each sampling point. The higher the WQI value, the better the water quality; conversely, the lower the WQI value, the worse the water quality. For rivers, it is more practical to determine reference and impaired points via comprehensive analyses of on-site investigation results and water quality. In conjunction with the field survey and WQI values, sampling sites S13-S17, which were less disturbed by anthropogenic activities near the reservoir, were used as the reference points in this study. The remaining sampling sites (S1–S12) were used as impaired points in the subsequent analysis.

**FIGURE 4:**
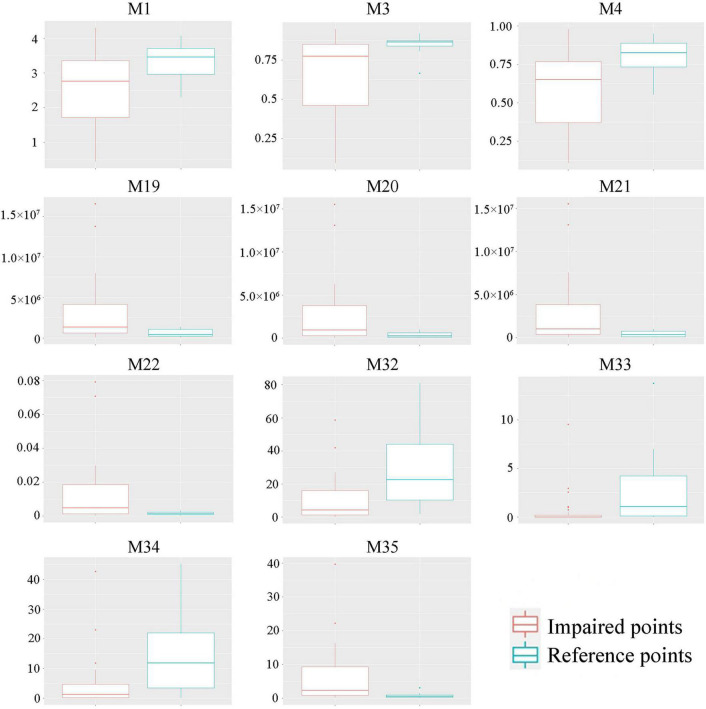
Boxplot of the selected indicators. The indicators are listed in Table 2.

First, we selected 35 candidate indicators that represented the diversity, abundance, biomass, and evenness of communities to establish the P-IBI evaluation system (Table 2). Second, indicators with little cabinet overlap were admissible to the subsequent screening stage; the metrics with the strongest separation power between the reference and impaired groups (the degree that the interquartile ranges overlapped between boxes, IQ < 2) were eliminated (Zhang et al., 2020). The 11 indicators with clear discrepancies between the impaired and reference points were selected for further analysis (Figure 4). Finally, using the Spearman correlation test (Supplementary Table 3), five non-redundant indicators were selected to calculate the P-IBI evaluation system: Shannon–Wiener index (M1), total biomass (M22), percentage of motile diatoms (M32), percentage of stipitate diatom (M33), and diatom quotient (M35).

**TABLE 2:** 35 candidate metrics.

Type of Metric	No.	Metrics
Diversity of	M1	Shannon-Wiener index
community	M2	Margalef index
	M3	Simpson index
	M4	Pielou index
	M5	Menhinick index
	M6	Odum index
	M7	Total number of taxa
	M8	Number of taxa of Bacillariophyta
	M9	Percentage of Bacillariophyta taxa
	M10	Number of taxa in Cyanophyta
	M11	Percentage of Cyanophyta taxa
	M12	Number of taxa in Chlorophyta
	M13	Percentage of Chlorophyta taxa
	M14	Number of non-diatom taxa
Abundance of	M15	Abundance of Bacillariophyta
community	M16	Abundance of Cyanophyta
	M17	Abundance of Chlorophyta
	M18	Mean Taxon Abundance
	M19	Total abundance
	M20	Abundance of top3 dominant species
	M21	Abundance of dominant species
Biomass of	M22	Total biomass
community	M23	Biomass of Bacillariophyta
	M24	Biomass of Cyanophyta
	M25	Biomass of Chlorophyta
Evenness of	M26	Percentage of abundance of Bacillariophyta
community	M27	Percentage of abundance of Cyanophyta
	M28	Percentage of abundance of Chlorophyta
	M29	Percentage of abundance of Chlorophyta and Bacillariophyta
	M30	Percentage of abundance of top3 dominant species
	M31	Percentage of abundance of dominant species
	M32	Percentage of motile diatoms
	M33	Percentage of stipitate diatom
	M34	Percentage of *Nitzschia*
	M35	Diatom quotient

The ratio method was used to calculate the P-IBI values at each site. Since the selected indicators M1, M32, and M33 all decreased as anthropogenic influence and interference increased, M22 and M35 had opposite response trends. The specific calculation methods according to the calculation formula are presented in Table 3. The P-IBI values were calculated using the aforementioned biological indicators, and the classification standard for the state of the aquatic environment was generated using the P-IBI. The 95% percentile (4.73) of the P-IBI for all sampling sites was the lower limit of Class I (excellent). P-IBI scores less than 4.73 were divided into four levels: “good” (II), “fair” (III), “poor” (IV), and “extremely poor” (V). Table 4 lists the classification criteria for ecosystem health.

**TABLE 3:** The ratio method’s calculation standard of 5 selected metrics.

No.	Selected indicators	Response to degradation	S
M1	Shannon-Wiener index	Decrease	(M1-0.45)/3.52
M22	Total biomass	Increase	(0.087-M22)/0.087
M32	Percentage of motile diatoms	Decrease	M32/54.84
M33	Percentage of stipitate diatom	Decrease	M33/6.69
M35	Diatom quotient	Increase	(39.68-M35)/39.54

**TABLE 4:** Based on the phytoplankton index of biotic integrity (P-IBI’s) grading standards of each ecosystem health status.

Grading	I	II	III	IV	V
Status	Excellent	Good	Fair	Poor	Extremely poor
Range	>4.73	[3.21, 4.73)	[2.76, 3.21)	[1.33, 2.76)	<1.33

### Ecosystem health assessments based on phytoplankton index of biotic integrity

**FIGURE 5:**
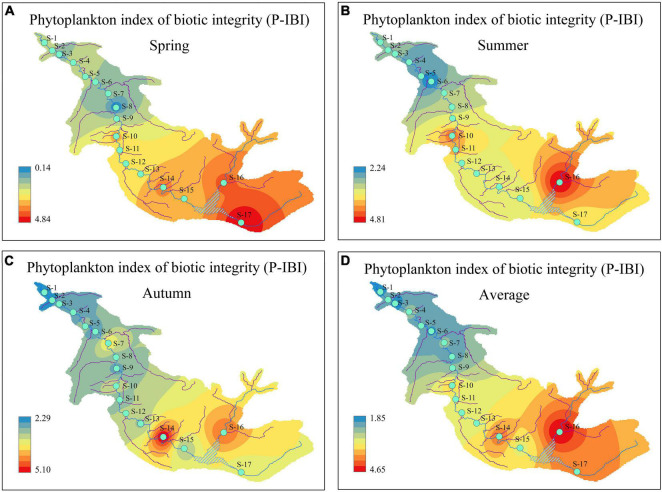
Temporal and spatial distribution of phytoplankton index of biotic integrity (P-IBI) during the study period. **(A)** spring; **(B)** summer; **(C)** autumn; and **(D)** average.

The health statuses of aquatic ecosystems at 17 sampling sites corresponding to the different seasons were classified according to the calculated P-IBI values and the classification standard in Table 4. From the perspective of seasonal change, the P-IBI values and health statuses in different seasons can be ranked as follows: summer (good) > autumn (fair) > spring (poor). Only the difference in the P-IBI values between spring and summer was significant (*P* < 0.01). Figure 5 illustrates that the health status of aquatic ecosystems had distinct spatial differences. In the present study, there was a difference of more than four evaluation levels from upstream to downstream of the river. The evaluation ranges for the mean P-IBI during the study period were “good” (4.65) to “poor” (1.85). The three sites of S10, S14, and S16-S17 were evaluated as “good”; S11-S13 and S15 were evaluated as “fair”; and the nine sites of S1-S9 were evaluated as “poor.”

### Multiple linear regression analysis between phytoplankton index of biotic integrity and environmental factors

On the basis of the significant correlations between the environmental factors and P-IBI, three environmental factors (Cond., pH, and DO) were screened to develop an MLR model. The eigenvalues, CI, variance proportion, tolerance, and VIF, which are characteristic parameters of the collinearity diagnostics, are listed in Table 5 and Supplementary Table 5. Multiple linear regression analysis showed that the regression equation was significant, *F* = 19.548, *P* < 0.001. Conductivity (*B* = -0.003, β = -0.441, *P* = 0.001), pH (*B* = -0.918, β = -0.656, *P* < 0.001), and DO (*B* = 0.151, β = 0.430, *P* = 0.005) significantly negatively and positively predicted P-IBI, respectively. Collectively, these variables explained 55.30% of the variation in P-IBI (Table 5), and the MLR model was as follows:


(5) P-IBI=8.887-0.003Cond. - 0.918pH+0.151DO

**TABLE 5:** The multiple linear regression of phytoplankton index of biotic integrity (P-IBI) and environment factors.

	Unstandardized Coefficients		Standardized Coefficients			Collinearity Statistics		Overall model		
Variable	*B*	Standard Error	β	*t*-test	*P*	Tolerance	VIF	*F*	R_*adj.*_^2^	Durbin-Watson
Constant	8.887	1.362		6.527	0			19.548***	0.553	2.085
Cond.	−0.003	0.001	−0.441	−3.639	0.001	0.678	1.475			
pH	−0.918	0.229	−0.656	−4.008	0	0.37	2.7			
DO	0.151	0.051	0.43	2.949	0.005	0.467	2.141			

VIF: variance inflation factor, ***P ≤ 0.001.

## Discussion

The IBI is a measurement method proposed by Karr (1981) that has evolved into the most widely used biological indicator. Many countries and regions have developed numerous IBI-based assessment methods for different levels of damage to aquatic ecosystems (Bae et al., 2010; Zalack et al., 2010; Wu et al., 2019). Phytoplankton comprise small individuals of a diverse range of species that occur in large quantities. They are extensively distributed in natural waters and constitute an essential component of river biodiversity; at the same time, they provide an important foundation for primary productivity in river ecosystems (Hötzel and Croome, 1999). Phytoplankton communities are the first response assemblage of organisms directly affected by environmental changes in aquatic lotic and lentic systems (Halsey and Jones, 2015). As rivers are dynamic and have expansive watersheds, the natural environment and socioeconomic conditions of the areas intersected by river flows are diverse (Acreman et al., 2014). The community structure of phytoplankton in rivers (lotic systems) is less stable than that of the phytoplankton in lakes and reservoirs (lentic systems), and both their seasonal changes are apparent (Tang et al., 2018; Minaudo et al., 2021). In addition, the biomass and species composition of phytoplankton in rivers are influenced by the combined effects of river morphology, hydrology, light, and reproduction rate, and they exhibit a clear spatial heterogeneity (Yang et al., 2019a). In recent years, the community structure of river phytoplankton has been influenced not only by the natural habitats of rivers but also by human activities and the upstream and downstream relationships of rivers (Zhao et al., 2020a). Human activities have gradually changed the types of land use in river basins, mainly through changes in nutrient enrichment, hydrological regimes, riparian habitat quality, and other ecological processes, resulting in a series of adverse effects on river ecosystems (Chen et al., 2022). With the escalation in anthropogenic stress, the loss of natural land, and the increase in pollutants discharged into rivers, the number of pollution-sensitive phytoplankton species in rivers decreased, and pollution-resistant species became the dominant group (Moghadam, 1975; Montoya-Moreno and Aguirre-Ramírez, 2013). The advantages of using phytoplankton as an IBI over other taxa (benthic diatoms, invertebrates, and fish) include simple collection, wide distribution, sensitivity to changes in aquatic conditions, short community renewal time, rapid response to changes in river water chemistry and habitat quality, and greater predictability of community trends (Zhou et al., 2019). Before and after a disturbance, the community structure of phytoplankton typically shifts dramatically, whereas the community structures of macroinvertebrates and fish are different (Wu et al., 2019; Feng et al., 2021). Therefore, phytoplankton can be used to assess the biological integrity of aquatic ecosystems, reflect the temporal and spatial changes in the condition of aquatic ecosystems in river basins, and serve as a crucial supplementary indicator for the evaluation of water quality.

Similar to the already adopted IBI based on fish and macroinvertebrates, our research has developed a standardized P-IBI system (Wu et al., 2019; Feng et al., 2021) to evaluate the ecosystem health status in the ASRB. The results showed that the aquatic ecosystem health status in the ASRB was “fair.” The evaluation based on P-IBI showed that the ASRB exhibited clear seasonal changes. According to Wu et al. (2012, 2019), the typical performance of the P-IBI evaluation system showed seasonal characteristics. According to the P-IBI results, the health of the aquatic ecosystem in the ASRB during summer was better than that during autumn and spring. This may be due to the fact that in temperate rivers, suitable temperatures, light, and nutrient conditions promote phytoplankton growth during summer (Wu and Zhang, 2013), thereby sustaining a high P-IBI value. From the perspective of the WQI, the seasonal change was autumn > spring > summer, indicating that the water quality was good in autumn and poor in summer during the study. According to the WQI classification, water quality in summer was the worst. This may be a result of exceptionally heavy rainfall in the area during the 2019 summer period, which resulted in domestic garbage entering the river via surface water runoff. Seasonal changes in nutrient concentrations are also important factors affecting P-IBIs and WQIs (Paerl et al., 2011; Song et al., 2017). Spring had substantially higher TN contents than those recorded in summer and autumn. The P-IBI indicated that the biological health of the phytoplankton community was at its worst in spring, while the WQI was higher than that in summer but remained at a “medium” level. Compared to other seasons, it was more common for a single phytoplankton species to dominate in spring. For example, *Cyclotella meneghiniana* Kützing had a higher degree of dominance at various sampling sites in spring. In addition, we found that the succession of dominant phytoplankton species exhibited substantial seasonal changes. In essence, diatoms bloomed in spring, Cryptophyta had a higher dominance in summer, and Chlorophyta and Cyanophyta dominated in autumn. Therefore, the P-IBI should be evaluated comprehensively in different seasons to eliminate evaluation errors caused by seasonal changes, which represents a more scientific approach for evaluating watershed health over a wide variety of time scales.

In this study, all reference points were rated as “good” or “fair” according to the P-IBI evaluation standard, whereas 16.7% of impaired points were rated as “fair” and 83.3% were rated as “poor.” These results indicated that P-IBI could acutely respond to environmental changes in the ASRB and accurately reflect the biological integrity at each sampling point (Supplementary Figure 1). Among them, the sampling points of “good” and “fair” were located in the upper reaches of the river, at the inlet and outlet of the Xiquanyan Reservoir, with high forest cover, shallow water, and high transparency, whereas the upstream current was swift, the phytoplankton abundance was low, and the area was less impacted by human activities. Sampling sites rated as “poor” were located downstream of the river in a dense urban area, with many sewage outfalls on both banks, slow flow, and turbid waters, and the area is strongly impacted by human activities. The P-IBI uses the ecological adaptability of different phytoplankton species to evaluate the health of river ecosystems. For example, the dominant phytoplankton species in the “fair” and “good” sampling sites included *Encyonema minutum* (Hilse) Mann, *Amphora ovalis* (Kützing) Kützing, *Gomphonema parvulum* (Kützing) Kützing, and *Navicula radiosa* Kützing. These dominant species are the most sensitive, and they have poor pollution tolerance; they cannot survive in heavily polluted waters. Therefore, their presence typically indicates a lightly polluted or non-polluted environment (Moghadam, 1975; Montoya-Moreno and Aguirre-Ramírez, 2013). The dominant species at the sampling sites with “poor” evaluation results mainly included *C. meneghiniana, Ulnaria ulna* (Kützing) Aboal, *Ankistrodesmus falcatus* (A. Braun) Korschikoff, and *Limnothrix redekei* Van Goor, which are common pollution-resistant taxa (Singh et al., 2013; Dwivedi and Srivastava, 2017). In addition, Zhang et al. (2019) determined from the P-IBI of the Bali River that pollution downstream of the river is the source of the lowest biological integrity in an estuary. The results of this study were consistent with our previous evaluation of the nutritional status in the ASRB using the benthic diatom index (Zhao et al., 2020b). In other words, rivers with obvious spatial changes in the ASRB were shown to be impacted by anthropogenic activities and nutritional conditions. This may be because there were more industrial activities in the middle and lower reaches of the ASRB, and the increase in sewage discharge caused by industrialization has caused a significant deterioration in downstream water quality (Li et al., 2018; Feng et al., 2019). Ultimately, this phenomenon affected the diversity and abundance of phytoplankton, thereby reducing the biological integrity index of phytoplankton in the lower reaches of the river. Consequently, the P-IBI could effectively reflect the health of the river ecosystem within the ASRB.

During the study, the P-IBI and WQI exhibited clear spatial changes during each season. Both indices showed a gradual decline, as anthropogenic activities increased from upstream to downstream of the ASRB. Wu et al. (2019) studied the Taihu Lake Basin and found that the value of the P-IBI gradually decreased as the degree of anthropogenic interference increased; concurrently, there was a positive correlation between the P-IBI and WQI (Supplementary Figure 2). This suggested that P-IBI and WQI were negatively correlated with anthropogenic interference, which was consistent with the results of this study. Comparing the evaluation results of the WQI and P-IBI, we found that both methods could distinguish the ecosystem health status of impaired and reference points, but that there were substantial variations in the evaluation results at the same sampling sites. At the same time, P-IBI and WQI exhibited seasonal characteristics. In previous studies, a single factor or indicator was typically used to evaluate water quality, and different results were obtained when different research methods were used. The WQI is weighted by the values of physical and chemical parameters, and the evaluation process is straightforward, quick, and simple to grasp; however, it lacks indicators related to the composition and structure of biological communities (De La Mora-Orozco et al., 2017; Nong et al., 2020). The P-IBI evaluation system lacked indicators for water quality, habitat, and hydrological conditions, and the evaluation process was relatively complicated. However, the P-IBI used phytoplankton as a biological indicator, and its community structure rapidly responded to habitat, hydrological conditions, and anthropogenic interference, and it integrated various indicators, such as community structure, biomass, and pollution tolerance. Therefore, P-IBI was a comprehensive tool that could accurately depict the health of river ecosystems. It compensated for the shortcomings of WQI when evaluating the health of rivers at the biological level. In this study, the P-IBI was constructed using a standardized method to effectively screen out indicators that are sensitive to anthropogenic disturbances. Furthermore, the current state of the aquatic ecosystem in the region could be derived through a comprehensive analysis of multiple indicators, which was a necessary complement to the water quality evaluation using WQI.

This study used environmental factors, such as WT, Cond., pH, DO, TP, COD_*Mn*_, TN, BOD_5_, and Tur. to perform multiple linear regression analyses with P-IBI. The results indicated that Cond. and pH values significantly negatively predicted P-IBI, whereas DO positively predicted P-IBI. Cond. was an effective indicator of the inorganic ion content in water. Studies have shown that the higher the water purity, the lower the Cond. value (Flores and Barone, 1998; Trebitz et al., 2019). In the waters of the ASRB, Cond. gradually increased from the upper to the lower reaches of the river. This might be due to the large amounts of industrial wastewater discharged into the middle and lower reaches of the river, increasing the inorganic ion content of the downstream water and making it less suitable for the growth and reproduction of phytoplankton. This phenomenon was consistent with the gradual decrease in P-IBI values from upstream to downstream. As an important ecological factor, pH was closely associated with phytoplankton growth. The pH of the water is primarily controlled by CO_2_ content; therefore, when the abundance of phytoplankton increases to a certain level, their biological activities have a certain impact on the CO_2_ content in the water, which in turn changes the pH of the water (Zhang et al., 2019). The P-IBI decreased as the pH increased in the ASRB. DO is a key factor influencing phytoplankton reproduction and metabolism. The decrease in DO concentrations has a negative impact on the growth of phytoplankton and its biomass (Xu et al., 2010). In this study, the DO concentration in spring was higher than that in autumn and summer. In terms of spatial changes, DO and P-IBI gradually decreased as anthropogenic activity increased. In addition, nitrogen is an essential nutrient in water, and it is a limiting factor in the growth of phytoplankton. An increase in TN can promote the reproduction of dominant phytoplankton species to a certain extent (Shetye et al., 2019), thereby changing the phytoplankton population structure. For example, the N/P mass ratios of sampling sites S3 and S8 in spring were close to the optimal N/P ratio for phytoplankton growth (7:1), (Hu, 2009), and the appropriate nutrient concentration promoted the propagation of a large number of the dominant species of *C. meneghiniana* (1.5 × 10^7^ ind./L and 1.3 × 10^7^ ind./L, respectively), resulting in the P-IBI values at these sites reaching their lowest values during the study period.

In this study, we developed a P-IBI system composed of various indices based on the multimetric indices concept. From a pool of 35 candidate ecological indices, the Shannon–Wiener index, total biomass, percentage of motile diatoms, percentage of stipitate diatom, and diatom quotient were selected based on their correlations and environmental factors. Wu et al. (2019) screened three indicators, phytoplankton density, chlorophyll a (Chl a), and Menhinick’s richness index, to create a P-IBI system to evaluate the ecological health of the Taihu Basin. Feng et al. (2021) suggested that the P-IBI be constructed based on the percentage of Cyanophyta genera, the number of total species, percentage of Cyanophyta abundance, Shannon–Wiener index, and Pielou index to evaluate the impact of anthropogenic activities (i.e., industrial activities, dam construction, and mining) on biological integrity. In addition, other studies have also considered the development and creation of indicators, such as the density of inedible algae, water-bloom-forming algal species biomass, and density of toxic phytoplankton (Yang et al., 2019b; Zuo et al., 2019). The IBI assessment system lacks comparability owing to a large number of candidate indicators, and the relationship between different indicators and the complexity of the health of different watersheds can be filtered. Studies evaluating the ecological health of rivers in northeast China based on P-IBI are sparse, and the results of this study provided crucial information on the biological integrity of the region.

Even though P-IBI was able to assess the ecosystem health of the ASRB, there were still some uncertainties in the development process. First, to develop the P-IBI, it was impossible to locate pristine water bodies that were completely unaffected by anthropogenic activities as reference points. Second, because P-IBI exhibited certain seasonal change characteristics, the sampling frequency should have been increased, and the river ecosystem health should have been assessed regularly. Third, the P-IBI should have been continuously verified using large quantities of data and adjusted accordingly to anticipate the future impact of anthropogenic activities on river ecosystem health. Future research should focus on these existing problems and should be conducted with more depth.

## Conclusion

The IBI is a potential tool for monitoring the ecological health of rivers, as it can reflect temporal and spatial changes in river ecological health. This study used the WQI and P-IBI to evaluate the impacts of anthropogenic activities on the ASRB. The seasonal characteristics of P-IBI were as follows: summer (good) > autumn (fair) > spring (poor). Based on the WQI evaluation, the water quality of the ASRB was best in autumn, followed by spring and summer. The overall health of ASRB was ‘fair’, and anthropogenic activities have severely damaged the biological integrity of the aquatic ecosystem in this region. Although the P-IBI and WQI established in this study showed similar spatial patterns and shared certain seasonal characteristics, the environmental state varied during different seasons. The development of P-IBI in ASRB enabled effective identification of the health status of reference and impaired points while compensating for the shortcomings of the WQI. Our findings have important implications for the ecological monitoring and protection of rivers impacted by anthropogenic activities.

## Data availability statement

The original contributions presented in this study are included in the article/Supplementary material, further inquiries can be directed to the corresponding authors.

## Author contributions

ZL was a major contributor to writing the manuscript, analyzing the data, and preparing figures and tables. XL and YF conceived and designed the experiments. ZL and CM performed the experiments. CM and YS contributed reagents and materials. All authors reviewed the manuscript.
