# Supplementary material for: Ecological health evaluation of rivers based on phytoplankton biological integrity index and water quality index on the impact of anthropogenic pollution: A case of Ashi River Basin
Source: Front Microbiol. 2022 Aug 26;13:942205. doi: 10.3389/fmicb.2022.942205 (PMC9459119; doi:10.3389/fmicb.2022.942205)
Supplement: Supplementary file 1 [file Data_Sheet_1.docx]

Supplementary Material

# Supplementary Figure


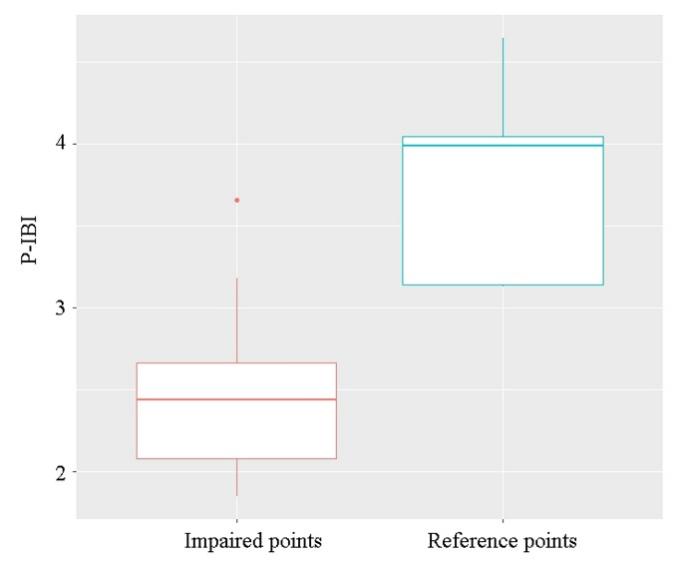


Figure S1 Boxplot of the phytoplankton index of biotic integrity (P-IBI).


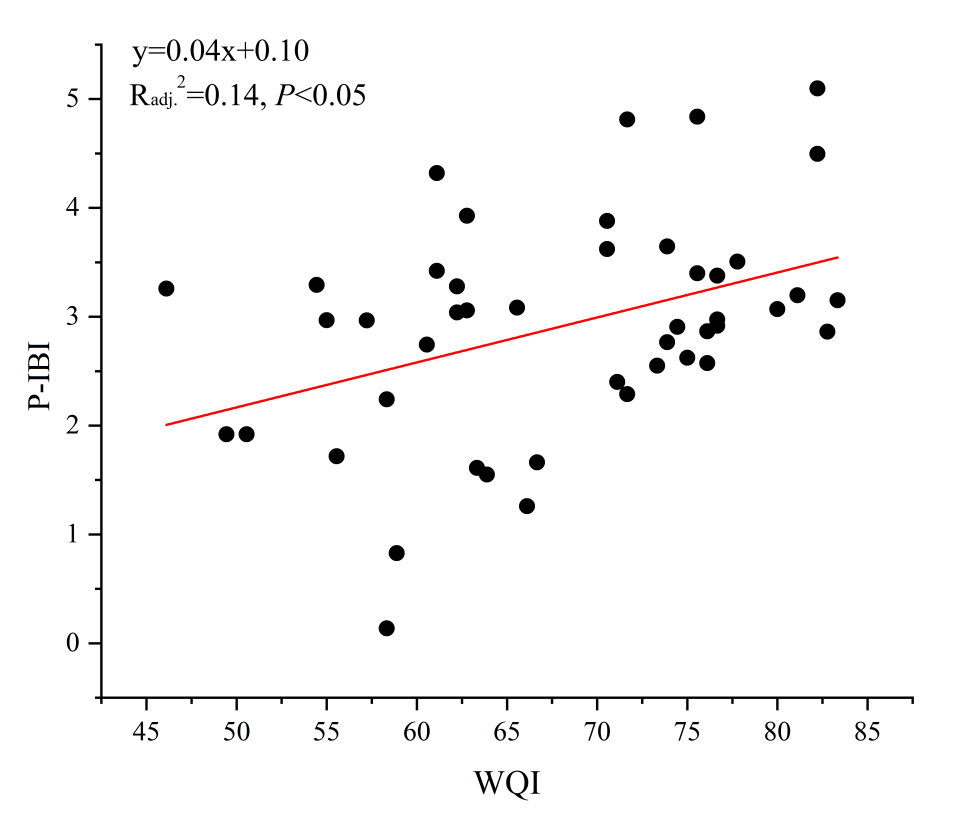


Figure S2 Relationship between the phytoplankton index of biotic integrity (P-IBI) and the water quality index (WQI) in the Ashi River Basin (ASRB).

# Supplementary Tables

| Table S1 Geographic location of sampling sites | | |
| --- | --- | --- |
| sites | latitude | longitude |
| S1 | 45°47′17″ | 126°44′55″ |
| S2 | 45°45′55″ | 126°46′9″ |
| S3 | 45°44′21″ | 126°47′45″ |
| S4 | 45°42′23″ | 126°52′18″ |
| S5 | 45°40′51″ | 126°53′38″ |
| S6 | 45°40′3″ | 126°54′31″ |
| S7 | 45°36′43″ | 126°58′10″ |
| S8 | 45°33′36″ | 126°59′52″ |
| S9 | 45°31′51″ | 126°59′55″ |
| S10 | 45°27′40″ | 127°0′13″ |
| S11 | 45°25′6″ | 127°0′42″ |
| S12 | 45°21′53″ | 127°1′55″ |
| S13 | 45°19′51″ | 127°5′13″ |
| S14 | 45°16′39″ | 127°10′29″ |
| S15 | 45°16′45″ | 127°10′07″ |
| S16 | 45°17′47″ | 127°22′49″ |
| S17 | 45°9′19″ | 127°26′28″ |

| Table S2 Weights and normalization factors of the parameters used in the calculation of the water quality index | | | | | | | | | | | | | | |
| --- | --- | --- | --- | --- | --- | --- | --- | --- | --- | --- | --- | --- | --- | --- |
| Variables | Abbreviation | Units | Relative | Normalization factor (Ci) | | | | | | | | | | |
|  |  |  | weight (pi) | 100 | 90 | 80 | 70 | 60 | 50 | 40 | 30 | 20 | 10 | 0 |
| Water Temperature | WT | °C | 1 | 21/16 | 22/15 | 24/14 | 12/26 | 10/28 | 5/30 | 32/0 | 36/−2 | 40/−4 | 45/−6 | >45/<−6 |
| pH | pH |  | 1 | 7 | 7-8 | 7-8.5 | 7-9 | 6.5-7 | 6-9.5 | 5-10 | 4-11 | 3-12 | 2-13 | 1-14 |
| Conductivity | Cond. | μS cm^-1^ | 1 | <750 | <1000 | <1250 | <1500 | <2000 | <2500 | <3000 | <5000 | <8000 | <12,000 | >12,000 |
| Turbidity | Tur | NTU | 2 | <5 | <10 | <15 | <20 | <25 | <30 | <40 | <60 | <80 | ≤100 | >100 |
| Dissolved oxygen | DO | mg L^-1^ | 4 | ≥7.5 | >7 | >6.5 | >6 | >5 | >4 | >3.5 | >3 | >2 | ≥1 | <1 |
| Total nitrogen^a^ | TN^a^ | mg L^-1^ | 2 | <0.1 | <0.2 | <0.35 | <0.5 | <0.75 | <1 | <1.25 | <1.5 | <1.75 | ≤2 | >2 |
| Total phosphorus^a^ | TP^a^ | mg L^-1^ | 1 | <0.01 | <0.02 | <0.05 | <0.1 | <0.15 | <0.2 | <0.25 | <0.3 | <0.35 | ≤0.4 | >100 |
| Permanganate index^b^ | COD_Mn_^b^ | mg L^-1^ | 3 | <1 | <2 | <3 | <4 | <6 | <8 | <10 | <12 | <14 | ≤15 | >15 |
| Biochemical oxygen demand for 5 days | BOD_5_ | mg L^-1^ | 3 | <0.5 | <2 | <3 | <4 | <5 | <6 | <8 | <10 | <12 | ≤15 | >15 |
| ^Adopted from Pesce and Wunderlin (2000) and Kannel et al. (2007)^ | | | | | | | | | | | | | | |
| ^a Normalization factors are according to the surface water quality standard GB3838-2002 (China), and weight as proposed by Kocer and Sevgili (2014).^ | | | | | | | | | | | | | | |
| ^b The range of normalized values and weight are according to Sun et al. (2016).^ | | | | | | | | | | | | | | |

| Table S3 Spearman correlation coefficient of metrics | | | | | | | | | | | |
| --- | --- | --- | --- | --- | --- | --- | --- | --- | --- | --- | --- |
|  | M1 | M3 | M4 | M19 | M20 | M21 | M22 | M32 | M33 | M34 | M35 |
| M1 | 1 | .939** | .782** | -.656** | -.722** | -.704** | -.687** | .336* | .457** | .376* | -.623** |
| M3 | .939** | 1 | .908** | -.774** | -.836** | -.811** | -.817** | .397** | .431** | .420** | -.706** |
| M4 | .782** | .908** | 1 | -.935** | -.965** | -.951** | -.952** | .462** | .334* | .495** | -.749** |
| M19 | -.656** | -.774** | -.935** | 1 | .980** | .989** | .981** | -.368* | -0.284 | -.401** | .647** |
| M20 | -.722** | -.836** | -.965** | .980** | 1 | .995** | .968** | -.421** | -.300* | -.451** | .691** |
| M21 | -.704** | -.811** | -.951** | .989** | .995** | 1 | .968** | -.384** | -.301* | -.417** | .658** |
| M22 | -.687** | -.817** | -.952** | .981** | .968** | .968** | 1 | -.348* | -.308* | -.390** | .668** |
| M32 | .336* | .397** | .462** | -.368* | -.421** | -.384** | -.348* | 1 | 0.278 | .901** | -.742** |
| M33 | .457** | .431** | .334* | -0.284 | -.300* | -.301* | -.308* | 0.278 | 1 | 0.261 | -.375* |
| M34 | .376* | .420** | .495** | -.401** | -.451** | -.417** | -.390** | .901** | 0.261 | 1 | -.742** |
| M35 | -.623** | -.706** | -.749** | .647** | .691** | .658** | .668** | -.742** | -.375* | -.742** | 1 |
| Note: ∗ ∗ indicated extremely significant correlation (P<0. 01);∗ indicated significant correlation (P<0. 05) | | | | | | | | | | | |

| Table S4 P-IBI values and ecosystem health status evaluation results | | | | | | | | |
| --- | --- | --- | --- | --- | --- | --- | --- | --- |
|  | Spring | | Summer | | Autumn | | Average | |
|  | P-IBI | Status | P-IBI | Status | P-IBI | Status | P-IBI | Status |
| S1 | 1.72 | Poor | - | - | 2.29 | Poor | 2 | Poor |
| S2 | 1.92 | Poor | 2.97 | Fair | 2.4 | Poor | 2.43 | Poor |
| S3 | 0.83 | Extremely poor | - | - | 2.87 | Fair | 1.85 | Poor |
| S4 | 1.92 | Poor | 2.97 | Fair | 2.77 | Fair | 2.55 | Poor |
| S5 | 1.61 | Poor | 2.5 | Poor | 2.92 | Fair | 2.26 | Poor |
| S6 | 1.55 | Poor | 2.5 | Poor | 2.57 | Poor | 2.06 | Poor |
| S7 | 1.26 | Extremely poor | 2.24 | Poor | 3.88 | Good | 2.46 | Poor |
| S8 | 0.14 | Extremely poor | 3.04 | Fair | 3.08 | Fair | 2.09 | Poor |
| S9 | 1.66 | Poor | 3.06 | Fair | 2.62 | Poor | 2.45 | Poor |
| S10 | 3.28 | Good | 4.32 | Good | 3.38 | Good | 3.66 | Good |
| S11 | 2.74 | Poor | 3.93 | Good | 2.86 | Fair | 3.18 | Fair |
| S12 | 2.55 | Poor | 3.26 | Good | 3.2 | Fair | 3 | Fair |
| S13 | 2.91 | Fair | 3.42 | Good | 3.07 | Fair | 3.13 | Fair |
| S14 | 3.65 | Good | 3.4 | Good | 5.1 | Excellent | 4.05 | Good |
| S15 | 2.97 | Fair | 3.29 | Good | 3.15 | Fair | 3.14 | Fair |
| S16 | - | - | 4.81 | Excellent | 4.5 | Good | 4.65 | Good |
| S17 | 4.84 | Excellent | 3.62 | Good | 3.51 | Good | 3.99 | Good |

Table S5 Collinearity statistics of the environmental factors derived from a multiple linear regression using a forwarding method.

|  |  |  |  | Variance Proportions | | | |
| --- | --- | --- | --- | --- | --- | --- | --- |
| Model | D | Eigenvalue | CI | Constant | Cond. | pH | DO |
| 1 | 1 | 1.814 | 1 | 0.09 | 0.09 |  |  |
|  | 2 | 0.186 | 3.122 | 0.91 | 0.91 |  |  |
| 2 | 1 | 2.769 | 1 | 0 | 0.03 | 0 |  |
|  | 2 | 0.228 | 3.489 | 0.01 | 0.7 | 0 |  |
|  | 3 | 0.003 | 30.004 | 0.99 | 0.28 | 1 |  |
| 3 | 1 | 3.729 | 1 | 0 | 0.01 | 0 | 0 |
|  | 2 | 0.24 | 3.939 | 0 | 0.74 | 0 | 0 |
|  | 3 | 0.029 | 11.356 | 0.05 | 0.05 | 0.01 | 0.6 |
|  | 4 | 0.002 | 43.599 | 0.95 | 0.2 | 0.99 | 0.39 |
| D denotes dimension; CI means the condition index | | | | | | | |
